# Molecular dynamics simulation of reversible electroporation with Martini force field

**DOI:** 10.1186/s12938-019-0743-1

**Published:** 2019-12-26

**Authors:** Cheng Zhou, Kefu Liu

**Affiliations:** Department of Light Sources & Illuminating Engineering, Fudan University, 220 Handan Road, Yangpu District, Shanghai, 200433 China

**Keywords:** Molecular dynamics simulation, Martini force field, Reversible electroporation, Phospholipid membrane

## Abstract

**Background:**

After the discovery of membrane-reversible electroporation decades ago, the procedure has been used extensively in biology, biotechnology and medicine. The research on the basic mechanism has increasingly attracted attention. Although most research has focused on models that consider all atomic and molecular interactions and much atomic-level information can be obtained, the huge computational demand limits the models to simulations of only a few nanometers on the spatial scale and a few nanoseconds on the time scale. In order to more comprehensively study the reversible electroporation mechanism of phospholipid membrane on the nanoscale and at longer time intervals of up to 100 ns, we developed a dipalmitoylphosphatidylcholine (DPPC) phospholipid membrane model with the coarse-grained Martini force field. The model was tested by separately examining the morphology of the phospholipid membrane, the hydrophilic channel size, the distribution of the voltage potential on both sides of the membrane, and the movement of water molecules and ions during electroporation.

**Results:**

The results showed that the process went through several stages: (1) the formation of the pore with defects originating on the surface. (2) The maintenance of the pore. The defects expanded to large pores and the size remains unchanged for several nanoseconds. (3) Pore healing stage due to self-assembly. Phospholipid membrane shrunk and the pore size decreased until completely closed. The pores were not circular in cross-section for most of the time and the potential difference across the membrane decreased dramatically after the pores formed, with almost no restoration of membrane integrity even when the pores started to close.

**Conclusions:**

The mechanism of the reversible electroporation process on the nanoscale level, including defects, expansion, stability, and pore closing stages on a longer time scale of up to 100 ns was demonstrated more comprehensively with the coarse-grained Martini force field, which took both the necessary molecular information and the calculation efficiency into account.

## Background

The discovery and utilization of cell membrane electroporation date from decades ago [1], and the research on the electroporation mechanism has increasingly attracted attention. Most of the current mechanistic studies are based on macroscopic and static models such as calculating the electric field distribution with the electric circuit model and the transmembrane voltage measured experimentally. However, as a non-equilibrium process on a molecular scale, the electroporation mechanism is determined by the movement of phospholipid molecules, water molecules and ions. Classical static and macro-models cannot describe unbalanced microscopic processes, such as the kinetics of pore formation, dynamics behavior of pores, as well as the transport of small ions across the pores in membrane. Therefore, in this paper, in order to explore the relationship between the movement of molecules and the reversible electroporation process, the molecular dynamics simulation software GROMACS was used to simulate the electroporation dynamics to reveal the basic mechanism on a molecular scale.

In the earliest experiments, it was found that the permeability of cell membranes could be enhanced by exposing cells to a suitable electric field [1]. The transient pores that formed in the membrane allowed the intracellular delivery of drugs, DNA, and other molecules. Chang et al. were the first to use cryo-electron microscopy to observe the formation of nanoscale (20–120 nm) pores on the surface of the cell membrane [2]. They showed that the electroporation process was inconsistent with electrical breakdown, because electrical breakdown involved chemical reactions.

The theory that first explained the electroporation process treated the phospholipid membrane as a flexible surface [3] that was squeezed under the influence of an electric field. If the electric field strength was sufficiently large to increase the compression force above a critical value, the phospholipid membrane could no longer withstand it and broke. This model did not take the molecular structure of the phospholipid membrane into account. New models proposed in recent years have added information about pore structure on the surface of phospholipid membranes [4, 5]. Some researchers considered electroporation as a phase transition since the structure of the phospholipid bilayer was metastable and the formation of pores could be considered the nucleation process of the other phase [6]. The electric field influences the nucleation rate and determines whether it will develop into a ‘crystallization’ process with irreversible electroporation. In the transient hydrophilic pore model, the phospholipid molecule itself was assumed to randomly generate some penetrating channels due to fluctuations in its thermal motion [4, 7]. When placed in a suitable electric field, the polar head of a phospholipid molecule is pushed toward the interior of a randomly generated tunnel, causing it to increase in length.

None of the above theoretical models took into account the effects of atomic motion. The method of molecular dynamics simulation is considered a powerful tool for elucidating the electroporation process. Molecular dynamics simulation has been used for decades as a research method, and has become a basic research tool in fields such as nanomaterials, bioengineering, and biochemistry. Tieleman et al. were the first to use all-atom molecular dynamics to simulate the electroporation process of phospholipid membranes [8]. They found that 3.04 ns after electric field application, the membrane underwent intense deformation and water molecules entered the membrane from both sides. When the water molecules from both sides met, pore channels were formed and the water molecules assumed a strong orientation within them. Tarek also used an all-atomic molecular dynamics simulation to study the electroporation process [9], and his simulations showed that under an electric field, water molecules on each side of the phospholipid membrane moved to the inside and when they met in the middle, hydrophilic pores were formed, and at the same time, the polar head of the phospholipid molecules tilted towards the hydrophilic pores. Bockmann et al. [10] reported that pore formation proceeded quickly within 0.5 ns once the process started and suggested a four-state pore formation model. Pore radius of 0.5 nm was then determined with 10 lipid headgroups tilting into the hydrophobic core forming a hydrophilic pore, which was in agreement with the experiment results. Polak et al. [11] found that electroporation threshold differed substantially, which depended not only on the “electrical” properties of the membrane, i.e., its dipole potential, but also on the properties of its component hydrophobic tails. Kotnik et al. [12] reported that without the strong electric field, the pore formation rate was generally too slow to be observable in such simulations, which typically covered a submicrosecond time span, but in sufficiently strong electric fields, the pore formation rate increases dramatically, and pore initiation was well discernible on a nanosecond time scale. Delemotte et al. [13] showed that at the molecular level, the external electric field and charge imbalance produced similar effects: provided the transmembrane voltage are higher than a certain threshold, hydrophilic pores stabilized by the membrane lipid headgroups form within the nanosecond time scale across the lipid core and both methods induced similar electric field distributions within the membrane core.

## Results

A dipalmitoylphosphatidylcholine (DPPC) phospholipid bilayer was established with ion concentration gradient across the membrane, and then the simulation was performed using the coarse-grained Martini force field. The model was tested by separately examining the pore morphology, the hydrophilic channel size, the distribution of the voltage potential across the membrane, and the movement of particles during electroporation.

### Morphology of the phospholipid membrane during electroporation

**Fig. 1:**
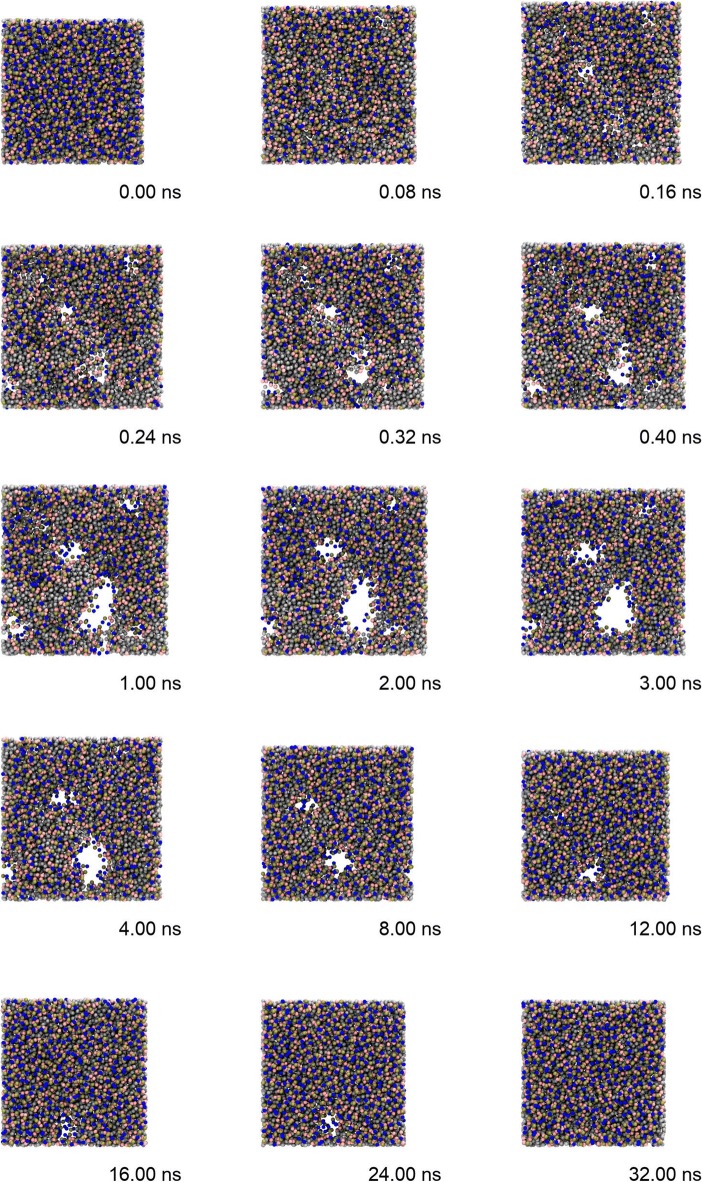
Morphological changes of phospholipids during electroporation within 32 ns. (1) 0–1 ns, initial stage with defects. (2) 1–4 ns, defects evolved into larger pores, and (3) 4–32 ns, pore healing stage

Figure 1 and the video (see Additional file 1) show the changes in the phospholipid membrane resulting from application of the electroporation field. Due to the presence of the ion concentration gradient, the phospholipid membrane was subjected to large surface tension and expanded in the initial stage (within 1 ns). As the phospholipid membrane’s volume was constant, the membrane inevitably thinned, which promoted pore formation. Several visible defects on the membrane surface appeared early, e.g., at 0.08 ns, and then gradually evolved into larger pores concomitant with the expansion of the phospholipid membrane. When the expansion process of the phospholipid membrane was complete (at about 1 ns), the pore size tended to be stable for several nanoseconds. After 4 ns, the phospholipid membrane shrank, and the pore size gradually decreased until they completely closed. Reformation of the whole complete membrane took around 30 ns.

### Size of hydrophilic pores

**Fig. 2:**
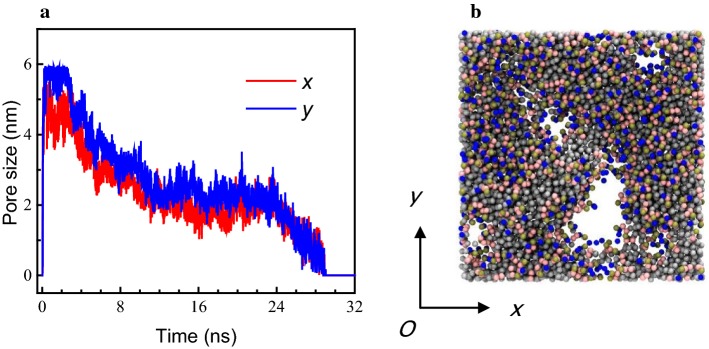
Sizes (nm) of hydrophilic pores. **a** Pore size in the *x* and *y* direction over time. **b** Molecule figure indicates the *x* and *y* direction. 0–1 ns, pores increased rapidly. 1–4 ns, pore size remained relatively constant. 4–30 ns pore size gradually decreased. After 30 ns pores all closed

Representative results are shown in Fig. 2. The dimensions of the pores in the *x*–*y* direction were measured to reflect their size and shape, since the pores were not ideal cylinders. From 0 to 1 ns, the pores increased rapidly from 0 to 6 nm (*y* direction) or 5 nm (*x* direction). Then, from 1 ns to 4 ns, the pore size remained relatively constant without much change. After 4 ns, the pore size in both directions gradually decreased, as well as the difference in diameter in the *x* and *y* directions, which implied that pores tended to become more circular. At 30 ns, the pores were all closed (0 nm).

### Microscopic process of pore formation

**Fig. 3:**
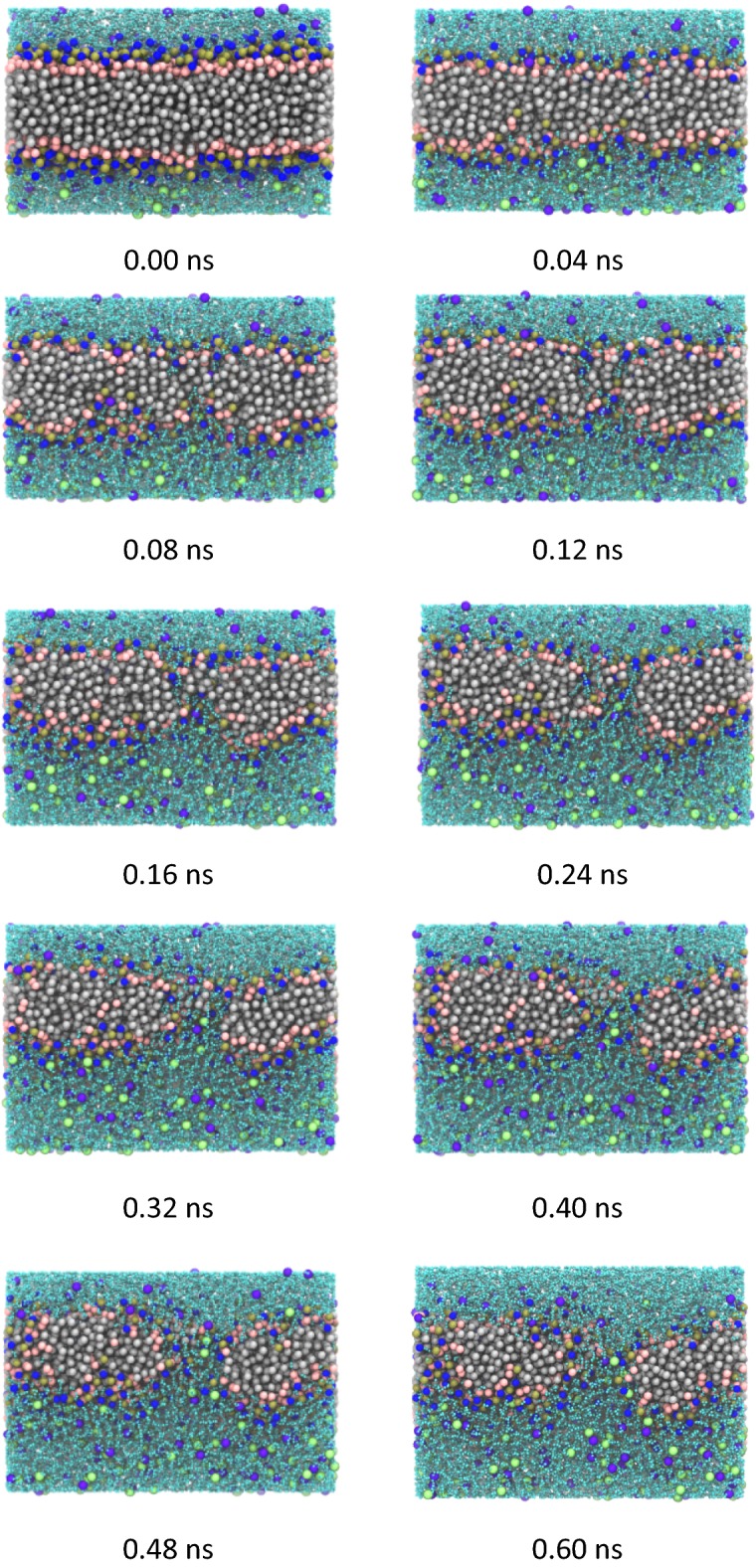
Microscopic process of pore formation (*color coding as in* Fig. 8). 0.04–0.08 ns, the hydrophobic region gradually exposed; 0.08–0.12 ns, the water chain formed with no obvious connectivity; 0.12–0.6 ns, water-chain pores completely formed

In order to further study the microscopic process of pore formation in the initial electroporation stage, here a pore was selected to observe the structure and molecular distribution near the pore. The results are illustrated in Fig. 3.

Due to the ion concentration gradients (namely electric potential difference) existing across the membrane, the hydrophobic region of the phospholipid was gradually exposed as the phospholipid membrane became thinner, and the probability of water molecules and ions entering the phospholipid hydrophobic region from both sides of the membrane gradually increased (0.04 to 0.08 ns). At 0.12 ns, water molecules from each side of the membrane met to form a chain of water molecules that almost penetrated the membrane. However, at this time the water chain was still incomplete with no obvious connectivity. At the same time, the hydrophilic heads of nearby phospholipid molecules tilted toward the water chain, which contributed to the energy required for the water molecule to contact the hydrophobic tail. The number of water molecules entering the hydrophobic region of the membrane was further increased and a more pronounced water chain was formed. In addition, ions also entered the phospholipid membrane through the water-chain pores, and ultimately passed through the membrane.

### Electrical potential distribution across the membrane

**Fig. 4:**
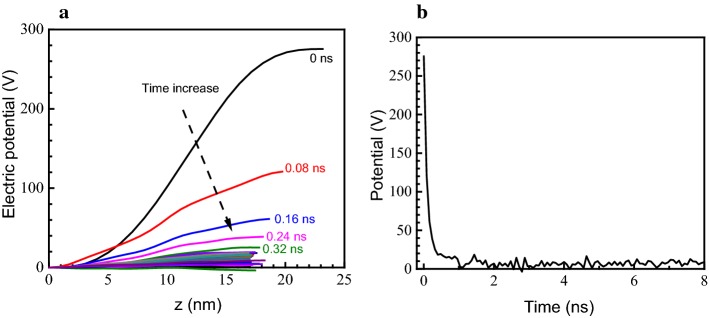
Distribution of electrical potential during the simulation. **a** Distribution of potential at different time in *z* direction; **b** transmembrane potential difference over time

The difference in electrical potential across the membrane reflected the stress imbalance on both sides, and therefore played a key role in the electroporation process. The distribution of the potential in the direction perpendicular to the phospholipid membrane (z direction) in the simulation box was calculated (Fig. 4a). Initially, the transmembrane potential difference was about 260 V, and the change in potential was located mainly in the phospholipid membrane and solution region, as the potential difference was caused by the distribution of ions. The potential difference then decreased over time. At 0.08 ns, the potential difference was reduced by more than half to around 100 V. The potential gradient at this time also decreased dramatically, indicating that the electric field force and the stress imbalance were much reduced. Afterwards, the potential difference and the slope of the potential decreased gradually, hence the electric field force was also greatly diminished. As can be seen in Fig. 4, during the initial stage of the electroporation simulation (0 to 0.5 ns), the potential difference rapidly decreased from 260 V to about 10 V. In subsequent simulations, the potential difference across the membrane fluctuated around this value but did not change much.

### Movement of water molecules and ions during electroporation

**Fig. 5:**
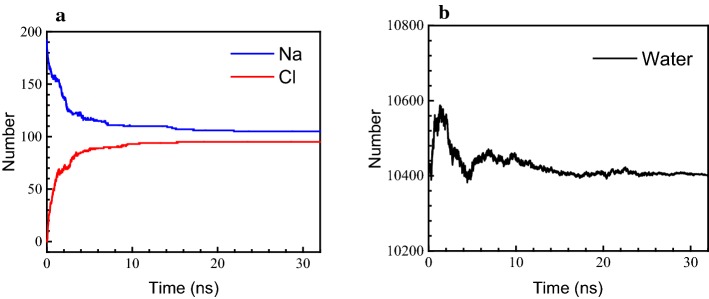
Number of particles over time in the upper compartment of the simulation box. **a** Sodium ions and chloride ions; **b** water molecules

The ion gradient causes the difference in membrane potential. The number of ions and water molecules in the upper compartment of the simulation box was calculated (Fig. 5) to determine the direct relationship between ion concentration and phospholipid morphology.

Initially, there were no Cl^−^ ions, 192 Na^+^ ions, and 10,434 water molecules in the upper compartment. In the initial stage (0 to 1 ns), the number of Cl^−^ ions increased rapidly while the number of Na^+^ ions decreased drastically, with the fastest ion migration speed. Because at this stage the pores were not yet formed completely, the transmembrane resistance for the ions was still high. However, as Fig. 4 shows, the potential difference across the membrane was also maximal during this stage. The extremely high electric field force during this time easily overcame the transmembrane resistance and caused the rapid migration of ions. In addition, the number of water molecules gradually increased, indicating that the direction of movement of the water molecules was consistent with that of the Cl^−^ ions.

In the second stage (1 to 4 ns), pores with a stable size formed. However, the ion migration speed was not higher than that in the first stage because the concentration difference across the phospholipids was already small. The potential difference across the membrane was likewise small, and the electric field force for driving ion diffusion was not significant (Fig. 4). This indicates that in the second stage, the migration of ions mainly depended on their concentration difference as they passed through the channels formed in the phospholipid membrane. In addition, the movement of the water molecules was not the same as in the first stage, as they returned to the lower compartment of the simulation box.

In the final stage, along with the gradual closure of the pores (4 ns), the ion migration speed was reduced to zero and these events are considered the most important features of this part of the process. At the same time, the diffusion rate of water molecules also decreased until it reached a steady state. At steady-state conditions, the number of positive and negative ions in the aqueous solution was not the same, which implied an unbalanced charge distribution across the membrane. However, as the imbalance in the ion distribution decreased, along with the difference in potential (Fig. 4), the driving force for ion transport became insufficient to overcome the transmembrane resistance of the closed or restored phospholipid membrane.

## Discussion

This MD simulation is aimed at investigating reversible electroporation in a long time-scale and providing a more comprehensive understanding of the mechanism, especially the healing stage. By using the coarse-grained Martini force field, we witnessed the process of (1) defects origination in the membrane within 1 ns, (2) water chain and pore channel formation with defects evolution, which lasts and keeps steady for around 3 ns, and (3) pore healing due to self-assembly until completely closed in the hydrophobic domain when subjected to an electrical field of maximum 10.5 V/nm. The whole process took about 30 ns. The overall results here presented are believed to capture the essence of the reversible electroporation in lipid bilayer. However, there are several limitations in this work should be noted:

1. We have a hypothesis here that applying an external electric field (*E*_ext_) can lead to an ion concentration difference. In the equilibrium state, there is no ion concentration difference across the phospholipid membrane. When an external electric field is applied, the equilibrium distribution is destroyed and the ions are unevenly distributed, which will form another electric field (*E*_ion_) on the membrane and affect electroporation process accordingly. Once the ion concentration difference is formed, *E*_ion_ will exist continually until the ion concentration difference disappears, no matter the external electric field (*E*_ext_) is removed or not. What we focused on in this simulation was the influence of the uneven ion concentration caused by *E*_ext_, namely an indirect method to deal with the effect of electric field.
  It is noteworthy that the electric potential difference or the electric field strength across the membrane was calculated based on the ion concentration, namely a double integral of the molecular charge density distribution. Although the electric field strength (10.5 V/nm) here is much higher than that commonly used in the experiment, it is essential in the MD simulation, because the increased ion concentration will not lead to significant distortion of electroporation process, and only by artificially increasing the field strength can speed up the electroporation process, which enables the whole process to be observed in a short and acceptable simulation time. Since the electric field *E*_ion_ is also much higher than *E*_ext_, the influence of the external electric field can be even neglected.
2. As this work is based on coarse-grained (not atomistically detailed) force field, it is preferred to analyze or understand the results in a qualitative or semiquantitative way rather than comparing specific values with other results. Nevertheless, results including pore formation time and pore size are even comparable with previous simulations [8–11] which adopted different models and initial conditions.
3. In this investigation, the simulation of reversible electroporation mechanism was performed with a non-reactive Martini force field, but in reality, the system involves chemical reactions [14–16] that cannot be simulated using this force field. In order to achieve this, a more accurate model is needed, including: (a) adopting all-atom force field directly with reactive force field. It is the most complex and accurate at the cost of computation efficiency. (b) In principle, introducing reaction field to Martini force field is possible and it is not likely to affect the molecule behavior very much. (c) Multi-scale simulation of a mixture of the all-atom and the coarse-grained model to accelerate the computation. Under the coarse-grained framework, higher precision simulations that reflect more molecular details can be implemented in the area we care about. However, the general theory and algorithms of multi-scale simulation have not yet been established at present. The simulation involving reaction needs to be extending by further study.

## Conclusions

The electroporation process in phospholipid bilayers was studied by coarse-grained molecular dynamics. Due to the application of an electric field, the equilibrium in the ion distribution across both sides of the membrane was destroyed. The ion concentration difference on each side caused a difference in electrical potential across the membrane. We simulated the electroporation process caused by this uneven ion distribution and divided the process into three stages:

(i) *Pore formation stage* When the phospholipid membrane was exposed to a difference in ion concentration, it first spread and the thickness decreased. At the same time, many defects appeared on the surface of the membrane, and these defects evolved into larger pores as the membrane extended. Under the setup conditions of the simulation in this study, the process occurred within the first nanosecond of electroporation. At this stage, water molecules entered the hydrophobic region of the phospholipid membrane from both sides and formed a water chain that communicated through the membrane’s pores. The ion migration was also faster, and the difference in ion concentration on each side of the phospholipid membrane decreased rapidly.
(ii) *Pore maintenance stage* This stage occurred after the formation of the channels, at about 1 ns. The size of the pores and the area and thickness of the phospholipid membrane remained stable, and this stage can last for several nanoseconds. Under the analog setup conditions of this study, the stage lasted approximately 3 ns. During this time, the ions could still undergo transmembrane migration, but the speed rapidly decreased.
(iii) *Pore healing stage* When the difference in ion concentration between the two sides of the phospholipid membrane was reduced, the area of the phospholipid membrane was also reduced. At the same time, the size of the pores gradually decreased until the pores were completely closed, and the phospholipid membrane was completely healed. This process can last for tens of nanoseconds. Under our simulation conditions, the process lasted from 3 to 30 ns. During this time, the transmembrane ion migration decreased until it stopped completely.

The overall results here presented are believed to provide a comprehensive mechanism of reversible electroporation. The adoption of coarse-grained Martini force field enabled the simulation to run in a long time-scale with relatively low computation cost. For chemical reaction involved, the model should be extended to be more accurate with reactive force field or the mixture of all-atom and coarse-grained force field. The findings and the results can provide the basis for the biomedical applications including cell apoptosis, drug and gene delivery.

## Methods

### Molecular dynamics simulations

Previous molecular dynamics simulations of electroporation were based on all-atom models. Although the all-atom model can yield atomic-level information about the process, the computational demand limits the simulation to only a few nanometers in distance and a few nanoseconds in time. In order to study the microscopic process of reversible electroporation more comprehensively, we conducted our study using a coarse-grained molecular dynamics simulation method. Before the molecular dynamics simulation was run, the energy of the system was minimized to ensure that the force on each atom was within an appropriate range to avoid the instability inherent in numerical integration. The algorithm implemented the steepest descent method, and the convergence criterion was that the force per particle was < 100.0 kJ mol^−1^ nm^−1^. The energy-minimized structure was used as the input for the simulation with time steps of 20 fs and periodic boundary conditions were applied. According to the standard treatment of the Martini force field, the Lennard-Jones effect was truncated at 1.2 nm and translated from 0.9 nm to 1.2 nm, ensuring force continuity. To calculate the static electric field, the particle mesh Ewald (PME) method was used [17] with a cutoff radius of 1.4 nm. The temperature was controlled at about 310 K by the Berendsen method [18] and the coupling constant was 1.0 ps. The Parrinello–Rahman method was adapted to control the horizontal and vertical pressure on the membrane at 1 bar, and the coupling constants were 10.0 ps, which gave a phospholipid membrane surface tension of 0 dyn/cm. The simulation was performed in an NVT ensemble.

### Model parameters

All the force field parameters in this study were from the Martini force field [19, 20], which is a coarse-grained (CG) force field that can take both the molecular information needed and the calculation efficiency into account. As it is not an atomistically detailed force field neglecting some atom degrees of freedom, sampling the energy landscape is considered as effectively as possible rather than as accurately as possible, which makes the Martini force field available to simulate a more complicated system and at longer time scale compared with all-atom force field. It has been widely used to study various structures and transformation process of phospholipid.

In the Martini force field, on average, an interaction center (namely a particle) represents four heavy atoms and associated hydrogen atoms. The interaction between particles is divided into two types: bonded and non-bonded interactions. Bonded interactions exist between particles within the same molecule, whereas non-bonded interactions between all particles. The systematical building-process of Martini force field combines “up-to-bottom” and “bottom-to-up” strategies. For the non-bonded interactions, the parameters are obtained by the reproduction of experimental partitioning free energies between polar and apolar solvents of a large number of chemical compounds (the “up-to-bottom” strategy); while the bonded interactions are derived from reference all-atom simulations (the “bottom-to-up” strategy).

The non-bonded interactions include van der Waals interactions and electrostatic interactions:

1 $$U_{\text{nonbonded}} = 4\varepsilon_{ij} \left[ {\left( {\frac{{\sigma_{ij} }}{{r_{ij} }}} \right)^{12} - \left( {\frac{{\sigma_{ij} }}{{r_{ij} }}} \right)^{6} } \right] + \frac{{q_{i} \,q_{j} }}{{4\pi \varepsilon_{0} r_{ij} }},$$

where $U_{\text{nonbonded}}$ is the non-bonded energy between two particles (*i ,j*), *ε*_*ij*_ and *σ*_*ij*_ are the intensity and distance of van der Waals interaction, *r*_*ij*_ is the distance of the particle pair, *q*_*i*_ is the charge of particle *i*, *ε*_0_ = 8.8542 × 10^−12^ C^2^ N^−1^m^−2^ is the dielectric constant in vacuum. In order to keep the model simple, only four main types of non-bonded interactions are defined: polar, non-polar, apolar, and charged. Each type has corresponding van der Waals interaction parameters.

The bonded interactions include the stretching, bending, and torsional terms, which are described by the following set of functions:

2 $$U_{\text{stretching}} = \frac{ 1}{ 2}k_{r} \left( {r_{ij} - r_{ij}^{0} } \right)^{2} ,$$

3 $$U_{\text{bending}} = \frac{ 1}{ 2}k_{\theta } \left( {\theta_{ijk} - \theta_{ijk}^{0} } \right)^{2} ,$$

4 $$U_{\text{torsional}} = \sum\limits_{n = 0}^{5} {C_{n} } \cos^{n} \left( {\phi_{ijkl} - 180^\circ } \right),$$

where $k_{r}$, $k_{\theta }$ and $C_{n}$ are the force constants, representing the intensity of each term; $r_{ij}$, $\theta_{ijk}$, $\phi_{ijkl}$ are the bond length, bond angle and dihedral angle between the atoms, respectively; $r_{ij}^{0}$ and $\theta_{ijk}^{0}$ are the equilibrium bond length and angle when the molecule is in the energy-minimized state.

**Fig. 6:**
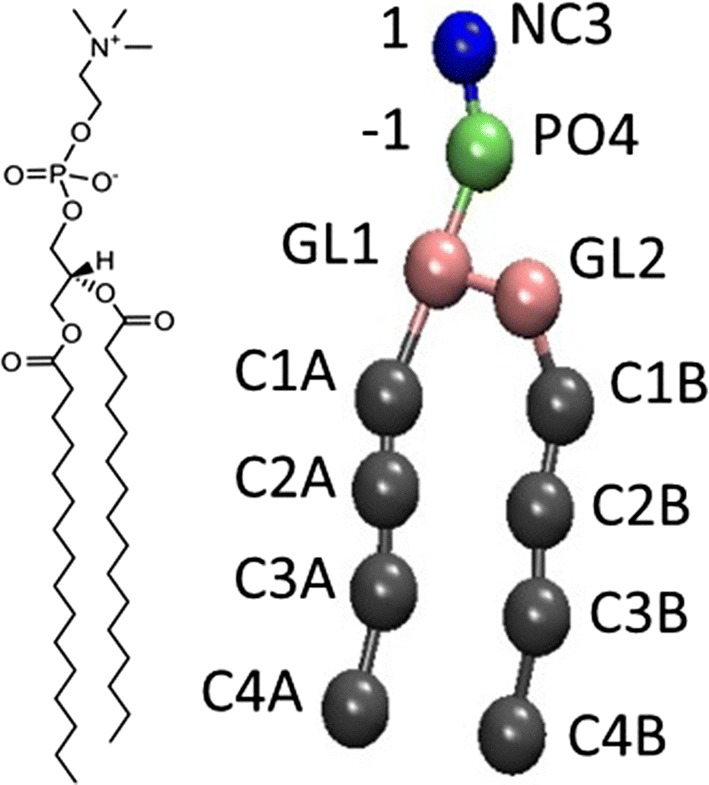
Molecular structure and coarse-grained model of 1,2-dipalmitoyl-sn-glycero-3-phosphocholine (DPPC). NC3: choline group, PO4: phosphate group. GL1 and GL2: glycerol skeleton. sn-1 (C1B–C4B) and sn-2 (C1A–C4A): alkyl chains

The phospholipid membrane was composed of 1,2-dipalmitoyl-sn-glycero-3-phosphocholine (DPPC) (Fig. 6). Every coarsely granulated DPPC molecule contained 12 Martini particles, each with a mass of 72 atomic mass unit (amu). The sn-1 and sn-2 alkyl chains consisted of four hydrophobic particles, labeled C1B–C4B and C1A–C4A, respectively. The polar head of DPPC consisted of two particles: a negatively charged PO4 particle representing a phosphate group, and a positively charged NC3 particle representing a choline group. The glycerol backbone was represented by two neutral particles (GL1 and GL2). GL1 linked the phosphate group and the sn-2 alkyl chain. GL2 linked GL1 and the sn-1 alkyl chain. A model with polarizable water molecules was adopted, which has proven to be suitable for studying electrostatic forces [21], including the electroporation process in this study.

### Establishing the model

**Fig. 7:**
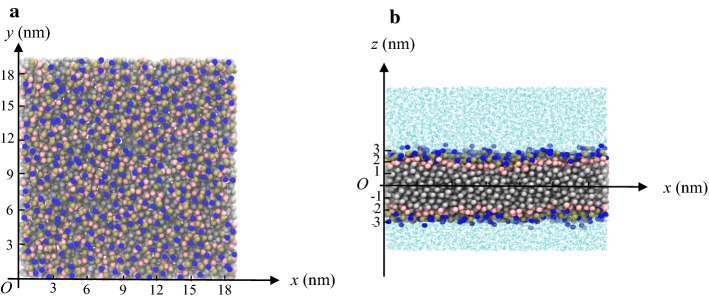
Diagram showing formation of the DPPC lipid bilayer. **a** Top view; **b** side view. In the figure, the NC3 particles (‘choline’) in the phospholipid molecule are shown in blue, the phosphate group particles (PO4) are yellow-brown, the neutral head of the phospholipid molecule (GL1, GL2) is pink and the hydrophobic tail particles are silver. The water molecules are blue-green

In order to construct the membrane, 512 phospholipid molecules and 10,477 water molecules were placed randomly and they formed a phospholipid bilayer due to self-assembly (Fig. 7). Then a 100-ns simulation was performed.

**Fig. 8:**
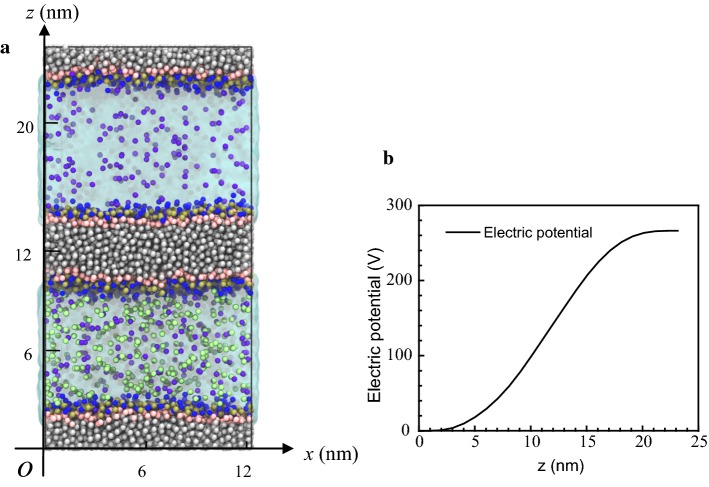
Setup for simulation of the electroporation process. **a** Side view of the simulation box; **b** distribution of the electric potential in the vertical direction. In **a**, the choline (NC3) particles in the phospholipid molecule are blue, the PO4 group particle is yellow-brown, the neutral head (GL1, GL2) is pink, and the hydrophobic tail particles are silver. The sodium ions are purple, the chloride ions green-yellow, and the water is blue-green

After forming the phospholipid membrane, a simulation box was constructed (Fig. 8). The entire simulation system consisted of two phospholipid membranes, each containing 512 phospholipid molecules. These two phospholipid membranes divided the aqueous solution into two regions, each contained 178 Na^+^ ions (corresponding to a physiological concentration of 0.15 M), but only the lower side contained 356 Cl^−^ ions. The entire simulation box was electrically neutral, but the solution had a net charge due to ion separation. This imbalanced ion distribution was formed by placing the phospholipid membrane in an electric field. The distribution of the electric potential in the vertical direction, perpendicular to the phospholipid membrane, is shown in Fig. 8b. It can be seen that the potential difference across the entire simulated box was about 260 V (the origin of the *z* axis was the lowest point of the box in Fig. 8a) and the electric field strength was approximately 10.5 V/nm. Since the periodic boundary condition (PBC) was adopted, the solution was connected with only one phospholipid bilayer in the simulation box. Therefore, our entire simulation system contained two phospholipid bilayers in order to obtain two unconnected solution regions.

### Structure of the self-assembled phospholipid bilayer

The phospholipid molecule will self-assemble to form a phospholipid bilayer structure once dissolved in aqueous solution, as the molecule is amphiphilic: the head is hydrophilic and the tail is hydrophobic. Figure 7 shows a typical bilayer structure in which the phospholipid molecules assembled into two layers. The molecules in one layer had the same orientation, while those in the other layer were oppositely oriented. This allowed the hydrophobic tail groups to associate with each other and avoid contact with water. The hydrophilic heads of the phospholipid molecules were distributed between the water and the hydrophobic tail region, in direct contact with water.

**Fig. 9:**
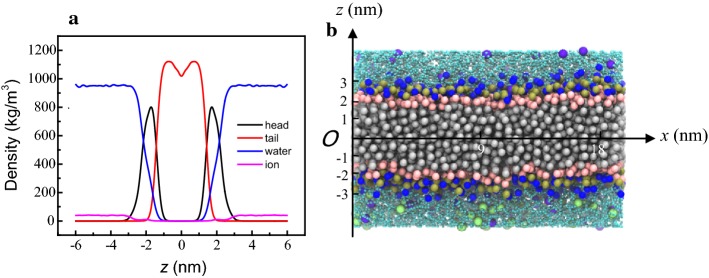
Density distribution of the self-assembled phospholipid structure. **a** Particle density in *z* direction. **b** Molecule figure indicates the *z* direction. The density distribution in the figure is translated so that in the middle of the phospholipid bilayer membrane, *z *= 0

Figure 9 shows the density distribution of different phospholipid components in the vertical direction, perpendicular to the bilayer. The hydrophobic tails of the phospholipid molecules were distributed between −2 and 2 nm, and the hydrophilic heads from −3 to −1 nm and from 1 to 3 nm. It can be seen that the thickness of the phospholipid membrane was 4–5 nm in the absence of external force. The density of water and ions gradually increased starting 1 nm from the middle of the phospholipid layer. Beyond 3 nm, water density reached the bulk maximum, while in the hydrophobic region, water density was zero, which means no water molecules entered the interior of the intact phospholipid bilayer. The energy barrier across the membrane was much too high for polar molecules such as water to overcome without the help of an additional force. Thus, electroporation of a phospholipid membrane can be regarded as a breaking down of the hydrophobic region by means of an electric field so that water or ions can easily cross the membrane.

### Hardware condition for simulation

The simulation process was mainly carried out on a Dell C6100 server with the open-source software, GROMACS 4.5.5 [22, 23]. The operating system was Red Hat Enterprise Linux Server release 5.6 (Tikanga). The main configuration of the computer node was 2*Intel(R) Xeon(R) CPU E5645@2.40 GHz 6-core processors, and 24 GB DDR3 1333 MHz memory.

## Supplementary information

**Additional file 1.** Morphological changes of phospholipids during the whole reversible electroporation process.

## Data Availability

All data generated or analyzed during this study are included in this published article, and the datasets used during the current study are available from the corresponding author on reasonable request.

## Abbreviations

DPPC: dipalmitoylphosphatidylcholine
PME: particle mesh Ewald
amu: atomic mass unit
PBC: periodic boundary condition
